# Sentience Matters: Analysing the Regulation of Calf-Roping in Australian Rodeos

**DOI:** 10.3390/ani12091071

**Published:** 2022-04-20

**Authors:** Morgan Stonebridge, Di Evans, Jane Kotzmann

**Affiliations:** 1 LLB (Hons), Research Assistant, Deakin Law School, Burwood, Melbourne, VIC 3125, Australia; m.stonebridge@deakin.edu.au; 2 Science Team, RSPCA Australia, Deakin, Canberra, ACT 2600, Australia; devans@rspca.org.au; 3 LLB (Hons), BCom, PGDipTeach(TFA), PhD, Alfred Deakin Postdoctoral Research Fellow, Deakin Law School, Burwood, Melbourne, VIC 3125, Australia

**Keywords:** sentience, calf-roping, welfare, regulation

## Abstract

**Simple Summary:**

The rodeo event of calf-roping, also known as the ‘rope and tie’, involves a contestant on horseback chasing, lassoing, catching, restraining, lifting and forcing a calf to the ground before tying the calf’s legs. The calves used in the event are handled in a manner that is likely to elicit fear and distress, and cause physical pain. In Australia, animal welfare legislation operates to prevent or constrain people from causing pain or distress to animals because animals are sentient beings. In this commentary, we discuss the research establishing that calves are sentient and explore the welfare concerns associated with calf-roping. We conclude that the concerns are such as to warrant protection under animal welfare legislation. The regulation of calf-roping in Australia is then analysed to explore how calves used in the event are excluded from this protection. We suggest that the exclusion of calves used in calf-roping from certain legal protections may be inconsistent with the purpose of animal welfare legislation, and that the variation in protection for these calves and other animals with corresponding levels of sentience requires further attention.

**Abstract:**

Animal sentience is recognised either implicitly or explicitly in legislation in all Australian states and territories. In these jurisdictions, animal welfare legislation prohibits acts of cruelty towards animals because animals have the capacity to experience pain or suffering. This acknowledgement is supported by scientific research that demonstrates animal sentience, as well as public opinion. Despite these legal prohibitions, calf-roping, a common event at rodeos, is permitted in the majority of Australian jurisdictions. In recent times, calf-roping has generated significant public concern due to the potential for injury, pain or distress for the calves involved. This concern is evidently shared in some overseas jurisdictions, such as New Zealand, where animal advocacy organisations have filed a legal challenge asserting that rodeo events violate New Zealand’s animal welfare legislation due to the pain and distress inflicted on the animals. This commentary discusses these welfare concerns, the legislative inconsistencies between Australian jurisdictions and the problematic legal status of calf-roping in Australia.

## 1. Introduction

Recent times have seen increasing public opposition towards rodeo events in Australia, most prominently towards the event of calf-roping [1,2]. This event, also referred to as the ‘rope and tie’, involves a competitor on horseback and a young calf, weighing on average only 100 kg and as young as four months of age. The calf is isolated from other calves when entering the chute. The competitor must chase the calf and throw a lasso around their neck, bringing the calf to a stop, before dismounting the horse, picking the calf up and forcing them to the ground. The competitor must then tie three of the calf’s legs together, which brings the recorded time to an end [3]. Competitors have 30 s to complete the rope and tie [3]; however, some competitors take longer, particularly where calves resist being caught. Moreover, many do not achieve a correct lasso around the neck or may not rope the calf at all.

Public opposition in relation to calf-roping is driven by the welfare risks for the calves used in the event. These concerns are largely formed on the basis of the calves’ sentience, which can be defined as their ability to feel or experience [4]. This ability extends to negative feelings, such as fear, pain and stress, as well as positive feelings such as contentedness and relief. Actions inherent to the event of calf-roping, such as the force a calf would feel when pushed to the ground and the impact on their neck when lassoed, give rise to welfare concerns due to their potential to elicit negative feelings—a concern supported by scientific research [5,6]. It is evident that calves attempt to flee from the contestant whilst being chased and try to escape after being lassoed, indicating a fear and stress response that is likely heightened by their isolation from other calves. Flight is a stress response designed to increase chances of survival. Additionally, calves are prey species, which may therefore limit their expression of pain and fear due to the evolutionary disadvantage of appearing weak or injured.

In this respect, animal welfare legislation enacted in all Australian states and territories seeks to protect animals from unnecessary harm because they can experience pain or suffering. While current scientific evidence demonstrates that calves are sentient and can experience physical and psychological pain and distress when used in a calf-roping event [5,6,7,8,9], they are largely excluded from the prohibition against causing unnecessary, unjustifiable or unreasonable harm to an animal contained in animal welfare legislation in all Australian jurisdictions. This commentary explores the welfare concerns for calves used in rope and tie events, the inconsistencies evident in the regulation of calf-roping in Australia and legal developments regarding calf-roping in New Zealand and Canada.

## 2. Calf Sentience

There is no universally accepted definition for the term sentience; however, the scientific community acknowledges that sentience encompasses the ability to have subjective experiences. This includes the ability to experience both positive and negative feelings, such as pain and pleasure [4,10]. There is also no objective measure for determining conclusively whether an animal—or human—experiences pain, due to the inherent subjectivity of the experience [10,11,12]. Conclusions as to sentience are typically drawn from behavioural or physiological responses to pain stimuli, such as attempts to escape, vocalisation or increased cortisol levels [13,14]. A recently published report by Birch et al., commissioned by the London School of Economics, identifies eight criteria for evaluating sentience, including the possession of nociceptors, integrative brain regions and connections between the two; responses affected by anaesthetics or analgesics and behaviour that indicates the animal values pain relief medication; behaviour indicating a balancing of threat as against reward; self-protective behaviour when subject to injury or threat; and associative learning that goes beyond habituation and sensitisation [13].

Based on scientific research, there is a consensus that most animals are sentient, and in particular that all vertebrates are sentient [4,10,12]. Thus, the current state of scientific understanding is clear that calves experience fear and physical and psychological pain, and are therefore sentient [5,6,7,8,9]. Accordingly, the event of calf-roping may inflict fear, pain and stress on the animals involved.

## 3. Welfare Concerns with Calf-Roping

Welfare risks can be assessed using the Five Domains Model, which considers and recognises subjective experiences of animals as being essential in evaluating overall welfare [15]. This model highlights the correlation between physical states and external situations which contribute to positive or negative experiences. By applying this model to assess the impacts of calf-roping on calves, it is clear that both physical and mental states are negatively affected. For example, calves may suffer physical injuries from the force of the rope against their neck or the impact their body makes with the ground, and may also experience psychological distress as a result of the constructed predator-prey scenario. In terms of physical pain, the calf may experience injury such as internal bruising, damage to their larynx and trachea, and broken bones [5,16]. Despite these welfare concerns, the Australian rodeo industry claims that all animals used in rodeo are unharmed and ‘treated like royalty’ [17]. This is supported by data collected by the Australian Professional Rodeo Association (APRA) through a survey of APRA affiliated rodeos, which gives an estimated injury rate of 0.072 per cent across all rodeo events, and for every ‘run’ an animal has in an event [17]. Injury rates specifically relating to calf-roping in Australia are not publicly available. It is relevant to note, however, that rodeos are not required to report animal injuries to a government department or any other relevant body in most Australian jurisdictions, and it is therefore possible that many of the injuries suffered by rodeo animals are not reported [18,19].

In this respect, the Professional Rodeo Cowboys Association (PRCA) in the United States claims an injury rate of approximately 0.00054% across all rodeo events, calculated using PRCA surveys [20,21]. However, accounts of animal welfare groups suggest that the injury rate is higher. For example, the Animal Legal Defense Fund filed a lawsuit against California Rodeo Salinas in 2014, claiming that animal injuries had been under-reported in contravention of California law [22]. In support, the animal welfare group Showing Animals Respect and Kindness had videotaped injuries to 41 animals over two years, while California Rodeo Salinas had only reported four during the same period [22]. The videos captured injuries such as calves unable to bear weight on one of their legs after being dragged to the ground, and a horse with a bloody wound to his neck the size of a tennis ball [22]. The animal welfare group consulted with a veterinarian concerning each of the suspected injuries, who opined that each of the injuries required veterinary treatment and therefore should have been reported. This is because section 4830.8 of the Business and Professions Code requires reporting for all animal injuries that occur at a rodeo event and need veterinary treatment [23]. These accounts indicate that it is entirely possible that rodeo animals suffer injuries in excess of that claimed by rodeo associations.

In addition, veterinarians are not required to attend rodeos or rodeo schools in most Australian states [19]. While veterinarians are required to be available for consultation, the lack of attendance inevitably results in some injuries being undetected and therefore unrecorded. Moreover, detecting bruising on dark coloured coated animals is extremely difficult [24]. Given the nature of the treatment of calves in rope-and-tie events, which involves the tightening of a rope around their necks and heavy contact with the ground, it is very likely that bruising occurs but that this is not visually obvious.

Fear and pain are significant threats to survival and elicit a stress response that may evoke the fight-flight-freeze system. As prey animals, calves are ‘less expressive’ of pain behaviour than humans are, as it is evolutionarily advantageous for them to feign strength to appear a more difficult target for predators [12]. Accordingly, indicators of any physical pain they experience during a calf-roping event may be subtle and easily missed. It should be noted that a lack of obvious outward expression of fear and pain does not provide evidence that calves who are chased and lassoed are not experiencing these negative feelings. Conversely, when a calf shows behavioural responses consistent with signs of fear and/or stress, this indicates a significant threat to both physical and mental state.

Against this backdrop, it is evident that calves also experience psychological harm as a result of calf-roping. Two recent Australian studies examining the impacts of roping on calves in rodeos demonstrate the harm that roping causes calves. A study by Sinclair et al. found that calves experienced with roping showed increased stress hormone levels following the repeated roping, and demonstrated behavioural reactions indicative of stress, including ‘white eye’ and attempts to flee [6]. The ‘white eye’ response, which is evident where the calf rolls their eye to reveal a 50% (or over) eye white to pupil ratio, occurred in 100% of the calves studied [6]. The study also considered the impact of roping on naïve calves’ stress hormone levels in the blood. They found an increase in stress hormone levels following roping, indicating an acute stress response in the calves [6].

A further study by Rizzuto et al. assessed the emotional state of calves during two phases of calf-roping using a qualitative behavioural assessment approach. Research participants were shown still images of calves being chased immediately after leaving the chute and calves leaving the arena after having the ropes removed, but were blinded to the context of the images [5]. They observed the calves being chased to be ‘more agitated, anxious, confused, energetic, frightened and stressed’ [5]. They considered the calves to be ‘more calm, contented, exhausted, inquisitive and relieved’ after release of the ropes [5]. These results indicate that roping is likely to elicit behaviour humans perceive as signifying fear, stress and anxiety in calves.

Thus, calf-roping clearly holds the potential to cause physical injury and there is evidence that it causes psychological harm to calves. Accordingly, the welfare concerns for calves used in calf-roping events are well founded. It might be reasonably expected, therefore, that animal welfare legislation—designed to protect animals against unnecessary harm—might recognise calves’ sentience and prevent this harm from occurring.

## 4. Recognition of Animal Sentience in Australia

Regulation of animal welfare in Australia is the responsibility of the states and territories, and all Australian states and territories have animal welfare legislation that prohibits acts that cause an animal unnecessary harm [25,26,27,28,29,30,31,32]. For instance, in Queensland, section 18 of the *Animal Care and Protection Act 2001* prohibits a person from being cruel to an animal, which includes causing an animal pain that is considered to be unreasonable in the circumstances [31]. Such legislation constitutes an implicit recognition of an animal’s capacity to suffer. The Australian Capital Territory goes a step further than other Australian jurisdictions by making recognition of animal sentience explicit. In the objects section of the *Animal Welfare Act 1992* (AWA), animals are recognised as ‘sentient beings that are able to subjectively feel and perceive the world around them’ [32]. The Act also acknowledges animals’ ‘intrinsic value’, and that they ‘deserve to be treated with compassion and have a quality of life that reflects their intrinsic value’ [32]. Victoria’s *Prevention of Cruelty to Animals Act 1986* is set to be replaced with a new Animal Welfare Act that is likely to explicitly recognise animal sentience in a manner similar to the Australian Capital Territory [33]. Further, Western Australia has recently undertaken a review of the *Animal Welfare Act 2002* (WA), which is expected to be amended to explicitly recognise animals as ‘living beings, able to perceive, feel, and have positive and negative experiences’ [34].

Legally recognising animal sentience, implicitly or explicitly, does not change the legal status of animals as property, although it might be perceived as differentiating between inanimate property and sentient property (animals) [35]. Even so, animals’ property status means that humans are still generally able to treat animals as they desire, subject to the protections provided by welfare legislation [35]. Explicitly recognising animal sentience does not create any direct legal obligations in relation to the care of an animal [35]. It may, however, impact the interpretation of animal welfare legislation by the courts. For example, in Australian states and territories that explicitly recognise animal sentience, a court determining whether a person has been cruel to an animal in violation of the relevant animal welfare legislation may need to consider whether that cruelty was ‘unjustifiable, unnecessary or unreasonable in the circumstances’ [32]. That consideration will be guided by the objects of the legislation, and accordingly, by the recognition that animals are sentient beings with intrinsic value. In turn, this may allow the courts to reach an interpretation that affords greater weight to the sentience and welfare interests of animals.

Explicit recognition of animal sentience in law clearly indicates that animals are provided protection against cruelty because of their capacity to experience pain and suffering. While this may seem an obvious conclusion, the acknowledgement of this premise raises questions about differing protections for animals with the same levels of sentience. For example, dogs and calves have commensurate levels of sentience [36]. However, when kept as companion animals, dogs are typically afforded the full protection of animal welfare legislation while calves are afforded only partial protection. This internal bias is illustrated by calf-roping, as the event would likely be in violation of animal welfare legislation in all Australian jurisdictions if dogs were used rather than calves. Such contradictions demonstrate the arbitrary nature of current animal welfare laws. However, expressly identifying scientific standards as providing a foundation for animal welfare legislation highlights these biases and may allow for strong arguments to be made against inconsistencies in protection for comparably sentient animals.

## 5. Regulation of Calf-Roping in Australia

As outlined, animal sentience is a quality that makes animals worthy of protection from harm. Despite this, calf-roping is permitted to occur in the majority of Australian jurisdictions.

In New South Wales, rodeos must be conducted in accordance with a specified rodeo code of practice [37]. Compliance with the NSW Code of Practice for Animals Used in Rodeo Events (NSW rodeo code) exempts rodeo operators from certain anti-cruelty provisions within the *Prevention of Cruelty to Animals Act 1979* (NSW *POCTA*), including sections 18 and 18 A. The NSW rodeo code explains that sections 18 and 18 A prohibits certain acts, ‘includ[ing] the use of cattle when part of an exhibition, spectacle or display where they could be cruelly treated or inflicted with pain and suffering’ [37]. As calf-roping is excluded from the requirement that calves not be cruelly treated, the extent of their welfare protection is instead covered by the NSW rodeo code. This code provides that all calves used in calf-roping events must weigh a minimum of 100 kg, which is industry standard, and must not be flipped over onto their back by the force of the rope once thrown around their neck [37]. Tasmania, Western Australia and the Northern Territory utilise similar frameworks.

In Tasmania, all rodeos conducted in accordance with the relevant code of practice are essentially exempt from the *Animal Welfare Act 1993*. The relevant code of practice in Tasmania is the ‘Standards for the Care and Treatment of Rodeo Livestock’, developed by the now defunct National Consultative Committee on Animal Welfare (NCCAW) [38,39,40]. The NCCAW standards were created to ‘set minimum requirements’ for the welfare of animals used in rodeo events [38]. As rodeos conducted in compliance with these standards are essentially exempt from Tasmania’s animal welfare legislation, rodeo operators are not subject to the prohibition against causing an animal unreasonable pain [25].

In Western Australia, the ‘Code of Practice for the Conduct of Rodeos in Western Australia’ (2003) (WA) is also based upon the NCCAW standards. Compliance with this code is not mandatory; however, section 25 of the *Animal Welfare Act 2002* (WA) provides a defence to a cruelty charge under section 19(1) if a person ‘was acting in accordance with a relevant code of practice’ [28]. The NCCAW standards have also been adopted in the Northern Territory by Gazette notice. The adopted standards operate in a similar manner to Western Australia, in that compliance is a defence to an animal cruelty charge [41].

Until recently, rodeo operators in Queensland were subject to the prohibition against causing an animal unreasonable harm. This is because Queensland was the only Australian jurisdiction that did not have regulations specific to rodeos. However, Queensland has now enacted a code of practice for rodeos that exempts operators and participants from the obligation not to cause an animal unreasonable harm should they comply with the provisions of the code [42]. The code sets minimum requirements that are largely in keeping with industry standards. While this is consistent with other Australian jurisdictions, the decision to codify existing practices may represent a missed opportunity for Queensland to bring the treatment of rodeo animals into line with community expectations—which is reflected to some degree in a petition calling for an end to calf-roping containing 60,000 signatures which was recently presented to the Queensland government [2].

Finally, Victoria, South Australia and the Australian Capital Territory do not permit calf-roping to occur. Victoria and South Australia prohibit the event by way of a minimum weight limit, as both states require that cattle used in calf-roping events weigh at least 200 kg [43,44]. APRA affiliated rodeos use cattle that weigh 200 kg or more for steer-roping as opposed to calf-roping [3], and one national rodeo association stipulates a maximum body weight of 140 kg for calves used in rope-and-tie events [45]. As such, the event is effectively prohibited in both Victoria and South Australia. In the Australian Capital Territory, however, calf-roping and rodeo more broadly are explicitly prohibited by section 18 of the AWA. It is therefore an offence to operate or participate in a rodeo in the Australian Capital Territory.

The above discussion highlights the discrepancies between state and territory regulation of calf-roping and underscores the need for a consistent approach. Sentience is largely accepted as the reason animal welfare matters and should therefore be the primary consideration in relation to determining which animals are to be protected. In this respect, animal welfare legislation in all Australian states and territories should aim to be consistent in relation to comparably sentient animals. This should be reflected in animal welfare legislation, and any variation from a level of protection that is commensurate with an animal’s sentience should require close scrutiny and exceptional circumstances.

## 6. Contemporary Developments

In light of the increased understanding of animal sentience and animal welfare, some developments concerning calf welfare are occurring in Australia and overseas. In Australia, all states and territories agreed upon the Australian Animal Welfare Standards and Guidelines for Cattle in 2016 (Cattle Standards) [46]. These standards will replace the existing codes of practice for cattle in an attempt to achieve national regulatory consistency. However, states and territories at present have failed to take a consistent approach to implementing these standards. For example, South Australia and Queensland have mandated the standards, whereas New South Wales has not mandated compliance [47]. Similarly, Western Australia has adopted the standards, but has not yet implemented the standards as regulations [47]. The Australian Capital Territory, Victoria, Tasmania and the Northern Territory are yet to implement the standards at all [47]. These standards may present an important development in terms of calf-roping, as they prohibit a person from dropping cattle ‘except to land and stand on their feet’ [46]. Also included as fundamental principles, are that handling equipment and procedures must minimise stress to the cattle and procedures must minimise the risk of pain or injury. Furthermore, those in charge of cattle have a responsibility to understand cattle behaviour and use low stress stock handling techniques and must minimise stress. Thus, the Cattle Standards, which ‘are based on current scientific knowledge, recommended industry practice and community expectations’ [46], contributes to the argument that chasing, lassoing and forcing a calf onto their side poses too great a risk to the welfare of the calf to be permitted. Given that these actions are inherent to calf-roping, this development appears to support a view that the practice of calf-roping is not aligned with the welfare interests of the animals involved nor best industry practice to handle calves safely and humanely.

Turning to developments overseas, two animal advocacy organizations have filed a legal challenge against the Minister of Agriculture and the National Animal Welfare Advisory Committee in New Zealand for failing to prohibit rodeo events [48]. The New Zealand Animal Law Association (NZALA) and Saving Animals From Exploitation (SAFE) argue that rodeo events are inconsistent with New Zealand’s *Animal Welfare Act 1999*, and therefore occur unlawfully in violation of that legislation [45]. This includes calf-roping, which NZALA and SAFE claim is one of the many rodeo events that does not ‘minimise the likelihood of unreasonable or unnecessary pain or distress’ for the animals involved [48,49]. This is an important development as the challenge will increase public awareness of the welfare concerns in relation to calf-roping, and may provide guidance to further legal reform in Australia.

An important development has also occurred in Canada, where law professor Alain Roy and his students filed an application for an injunction to prevent a rodeo in Montréal from going ahead in 2017 [16]. The application was withdrawn and an out of court agreement was reached; however, as part of this agreement the plaintiff was granted unlimited access to two rodeos to collate evidence on the welfare of the animals [50]. This resulted in a comprehensive report that provides recorded observations of the welfare impact on rodeo animals, including calves used in calf-roping events. For example, a veterinary anaesthesiologist who analysed the footage detailed the negative physical impact on calves thrown to the ground, explaining that such actions can create ‘a risk associated with the sudden increase in intrathoracic pressure upon contact with the ground, which can cause damage’ to the animals [16]. As calf-roping is conducted in a similar manner in Canada and Australia, the observations contained in this report provide valuable insight into the welfare of calves in Australia, and contribute to the body of evidence suggesting that the existing legal protections for calves in Australia are not commensurate with their sentience.

## 7. Conclusions

This discussion has demonstrated that sentience forms the primary basis of animal protection in Australia. Animal welfare legislation in all Australian jurisdictions recognises either implicitly or explicitly that animals are in need of protection because of their capacity to experience negative feelings and therefore their capacity to suffer. Accordingly, the scientific consensus that calves are sentient indicates that their welfare should be protected. Currently, calf-roping events are permitted to occur in all Australian jurisdictions except Victoria, South Australia and the Australian Capital Territory. This is despite evidence that demonstrates the inherent risks posed to the welfare of calves in calf-roping events. The potential legal issues with calf-roping are highlighted by developments in Australia and overseas, which suggest that calf-roping events force calves to experience negative mental states and risk physical injury, and, accordingly, do not meet their welfare requirements. Ultimately, where sentience is accepted as the primary purpose for preventing unreasonable and unnecessary harm to animals, the continued use of young calves in rope-and-tie events appears to be inconsistent with the purpose of animal welfare legislation and must raise questions about the adequacy of legal protection.

## Data Availability

Not applicable.
